# Altered synaptic homeostasis: a key factor in the pathophysiology of depression

**DOI:** 10.1186/s13578-025-01369-y

**Published:** 2025-02-25

**Authors:** Bokai Wang, Teng He, Guofan Qiu, Chong Li, Song Xue, Yuanjia Zheng, Taiyi Wang, Yucen Xia, Lin Yao, Jinglan Yan, Yongjun Chen

**Affiliations:** 1 Institute of Acupuncture and Moxibustion, Shandong University of Traditional Chinese Medicine, 4655 University Road, Jinan, 250355 China; 2 Chinese Medicine Innovation Research Institute, Shandong University of Traditional Chinese Medicine, Jinan, 250355 China; 3 Key Laboratory of Traditional Chinese Medicine Classical Theory, Ministry of Education, Shandong University of Traditional Chinese Medicine, Jinan, 250355 People’s Republic of China; 4 Shandong Key Laboratory of Innovation and Application Research in Basic Theory of Traditional Chinese Medicine, Shandong University of Traditional Chinese Medicine, Jinan, 250355 China

**Keywords:** Depression, Synaptic homeostasis, Ion channels, Presynaptic neurotransmitter release, Synaptic scaling, BDNF, TNF-α, Retinoic acid

## Abstract

Depression, a widespread psychiatric disorder, is characterized by a diverse array of symptoms such as melancholic mood and anhedonia, imposing a significant burden on both society and individuals. Despite extensive research into the neurobiological foundations of depression, a complete understanding of its complex mechanisms is yet to be attained, and targeted therapeutic interventions remain under development. Synaptic homeostasis, a compensatory feedback mechanism, involves neurons adjusting synaptic strength by regulating pre- or postsynaptic processes. Recent advancements in depression research reveal a crucial association between the disorder and disruptions in synaptic homeostasis within neural regions and circuits pivotal for emotional and cognitive functions. This paper explores the mechanisms governing synaptic homeostasis in depression, focusing on the role of ion channels, the regulation of presynaptic neurotransmitter release, synaptic scaling processes, and essential signaling molecules. By mapping new pathways in the study of synaptic homeostasis as it pertains to depression, this research aims to provide valuable insights for identifying novel therapeutic targets for more effective antidepressant treatments.

## Introduction

Depressive disorder, a pervasive mental health condition, is primarily characterized by low mood, anhedonia, lack of motivation, and social deficits. Affecting over 280 million people worldwide [1, 2], it imposes a significant burden on society and individuals alike. Although pharmacological treatments are widely used for managing depressive disorder, they often come with side effects and limitations, such as delayed onset of action, high relapse rates, headaches, sleep disturbances, gastrointestinal issues, sexual dysfunction, withdrawal symptoms, and an increased risk of suicidal ideation. Moreover, these treatments are ineffective for a subset of patients [3–5].

Synaptic homeostasis, a crucial compensatory negative feedback mechanism, involves neurons adjusting synaptic strength by regulating presynaptic neurotransmitter release and the expression or localization of ion channels or neurotransmitter receptors on the postsynaptic membrane, thereby counteracting excessive excitation or inhibition [6–9]. This process operates over hours to days, restoring neurons to their set point and maintaining neural network stability. Recent research has increasingly linked synaptic homeostasis to a range of neuropsychiatric disorders, including autism spectrum disorders, Parkinson’s disease, Alzheimer’s disease, epilepsy, and schizophrenia [10–14].

It has been proposed that depression may stem from disruptions in the homeostatic mechanisms regulating synaptic plasticity [15]. However, the exact pathways linking synaptic homeostasis to depression are not yet fully understood. As research continues to explore the neurobiological underpinnings of depression, the complex relationship between the disorder and synaptic homeostasis is becoming clearer. This review aims to examine the correlation between depression and imbalances in synaptic homeostasis, as well as the potential antidepressant mechanisms of medications that modulate synaptic homeostasis, with the goal of advancing both research and therapeutic strategies for depression.

## Synaptic homeostasis as a critical factor in depression

**Fig. 1:**
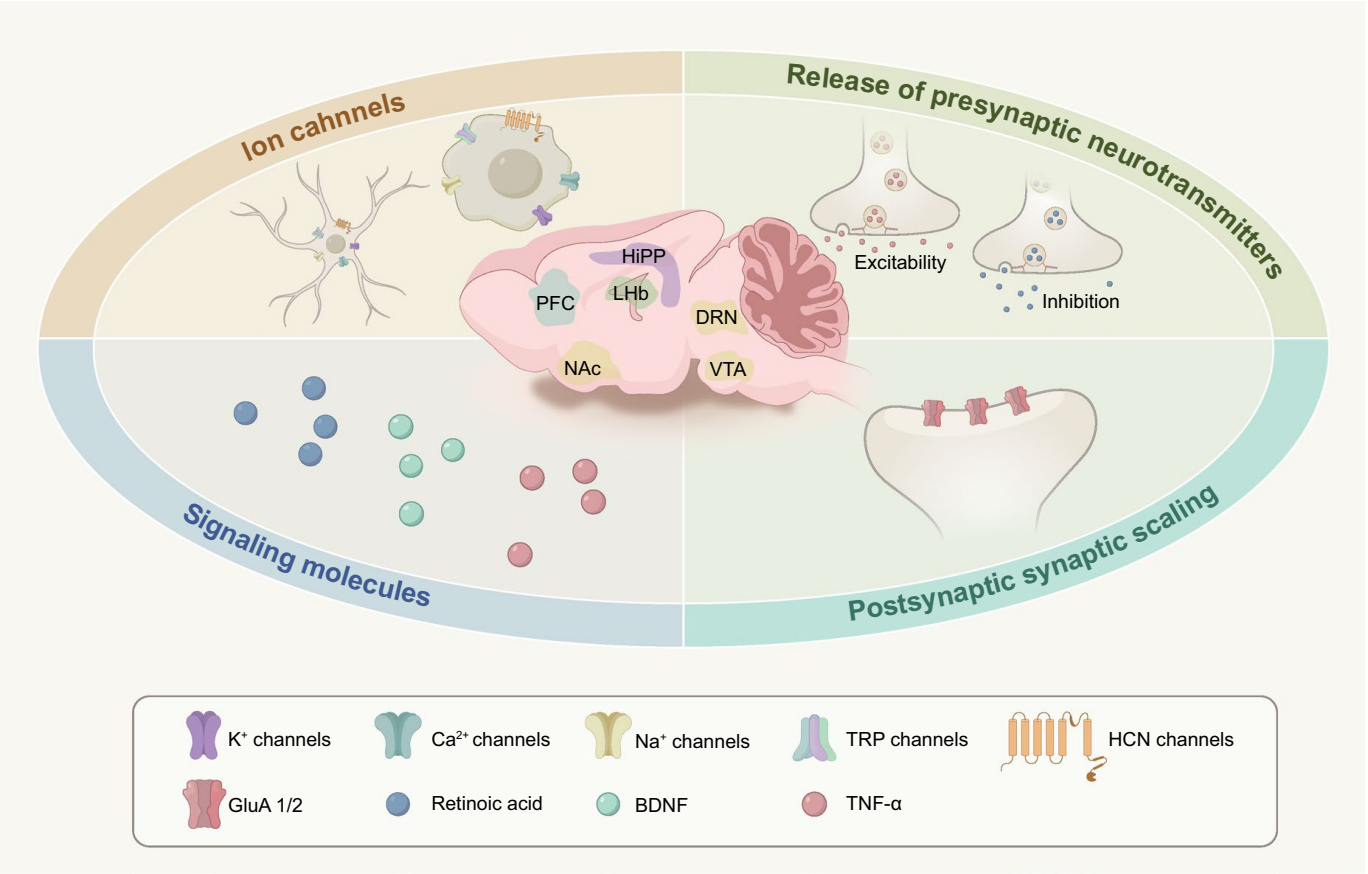
Synaptic homeostasis plays an important role in the pathogenesis and treatment of depression. Several mechanisms of synaptic homeostasis are critically involved in multiple brain regions during the onset and treatment of depression

Synapses, the specialized intercellular junctions between neurons or between neurons and other cells, are essential for transmitting information via electrical and chemical signals, thereby constituting the fundamental unit of communication within neural networks [16, 17]. As critical components of the central nervous system (CNS), synapses are particularly susceptible to various stimuli. Stress and depression can result in the reduction of brain region volumes, such as the prefrontal cortex (PFC) and hippocampus, which are pivotal for mood and cognition [18–22], and can also lead to a decline in the number and function of dendritic spines [23–27]. Antidepressant treatments have demonstrated the ability to reverse these adverse changes [28–31], underscoring a significant link between synaptic dysfunction and depressive states. The preservation of normal synaptic function is dependent on homeostatic mechanisms that stabilize synaptic transmission amidst fluctuating conditions. These mechanisms typically encompass (1) ion channels [32–34], (2) presynaptic neurotransmitter release [8, 35], (3) synaptic scaling of the postsynaptic density [36], and (4) related signaling molecules [37, 38]. Mounting evidence indicates that disturbances in synaptic homeostasis are integral to the mood-related circuitry disruptions observed in depression (Fig. 1).

## Ion channels in synaptic homeostasis: implications for the pathophysiology of depression

Alterations in intrinsic neuronal excitability, a hallmark of various CNS diseases [39], are regulated by the properties, distribution, and abundance of ion channels embedded in the cell membrane. These channels are crucial for converting synaptic inputs into specific neuronal outputs [40]. Ion channels, including voltage-gated calcium [41, 42], potassium [43, 44], and sodium channels, facilitate ion exchange across the cell membrane, thereby maintaining the balance between neuronal excitation and inhibition. They directly modulate neurotransmitter release and synaptic efficacy, and indirectly influence neuronal excitability. The interplay among these channels generates action potentials, regulates neuronal firing frequency, and affects synaptic homeostasis by modulating synaptic transmission. Disruptions in ion channel function can significantly impact the CNS, and such imbalances are implicated in the development of psychiatric disorders such as bipolar affective disorder, autism, epilepsy, schizophrenia, and depression [45].

Ion channel alterations within the CNS have been documented in both depressive patients and animal models, as summarized in Table 1. For instance, in the striatal-nucleus accumbens of patients with major depressive disorder (MDD), there is an upregulation of genes encoding voltage-gated potassium, calcium, and sodium channels, except for the voltage-gated potassium channel (K_V_) 9.3 [46]. Conversely, down-regulation of *KCNJ10* mRNA (encoding inwardly rectifying potassium channel (Kir)2q14.1 in the hippocampus) and *SCN1A* mRNA (encoding voltage-gated sodium channel (Na_V)_ 1.1 in the PFC) has been observed in MDD patients [47, 48]. Enhanced expression of Kir4.1 and γ-aminobutyric acid (GABA) B receptor subunit 1 proteins is detected in the parietal cortex of MDD patients [49], along with elevated transient receptor potential melastatin 2 (TRPM2) protein expression in the hippocampus [50].

**Table 1:** Alterations of Ion Channels in Various Brain Regions Associated with Depression

Channel Types	Research Subjects	Brain region	Change	References
Kir4.1	MDD patients	Parietal cortex	Increase	[49]
*KCNJ10*(Kir4.1)	MDD patients	Hippocampus	Decrease	[47]
*SCN1A*(Na_V_1.1)	MDD patients	PFC	Decrease	[48]
TRPM2	MDD patients	Hippocampus	Increase	[50]
K_V_2.1	CMS rats	FC	Increase	[52]
*KCNB1*(K_V_2.1)	aLH mice	LHb	Increase	[51]
K_V_3.1	CMS rats	Hippocampus	Decrease	[52]
K_V_4.2	CMS rats	FC and hippocampus	Decrease	[52]
K_V_4.2	CSDS mice	LHA GABAergic neuron	Decrease	[53]
K_V_4.2	CUMS mice	NAc medium spiny neurons	Function Degradation	[55]
K_V_7.4	CSDS mice	VTA dopaminergic neurons	Decrease	[54]
*KCNJ9*(Kir3.3)	WKY rats	MHb	Increase	[59]
*KCNJ5*(Kir3.4)	WKY rats	LHb	Increase	[59]
Kir4.1	cLH rats and LPS rats	LHb	Increase	[57]
Kir4.1	LPS treated mice	Hippocampus	Increase	[58]
Kir6.1	CUMS mice	Hippocampus	Increase	[61]
Kir6.1	CUMS mice	mPFC	No change	[61]
Kir6.1	CMS mice	Hippocampus	Increase	[60]
Kir6.2	CMS mice	Hippocampus	Increase	[60]
SK3	CSI mice	DRN	Increase	[76]
TREK1	CMS rats	FC	Increase	[52]
TREK1	CUMS mice	Hippocampus	Increase	[64]
TREK1	LPS rats	Hippocampus	Increase	[65]
CaV1.2	CRS rats	Hippocampus	Increase	[67]
CaV1.2	CUMS mice	PFC	Increase	[68]
CaV1.2	CUMS mice	Dorsal hippocampus	No change	[68]
CaV1.2	CUMS mice	Ventral hippocampus	No change	[68]
CaV1.2	CUMS mice	Amygdala	No change	[68]
HCN1	CUS rats	Dorsal CA1	Increase	[77]
HCN2	SNI mice	LHb	Increase	[78]
HCN2	CMS mice	VTA	Decrease	[71]
HCN2	SDS mice	NAc Shell Cholinergic interneurons	Decrease	[72]
TRPV2	CUMS rats	Hippocampus	Decrease	[74]
TRPV4	LPS mice	Hippocampus	Increase	[75]
TRPM2	CUS mice	Hippocampus	Increase	[50]

Kir: inwardly rectifying potassium channel; Na_V_: voltage-gated sodium channel; K_V_: voltage-gated potassium channel; SK3:small conductance calcium-activated channel 3; TREK: TWIK-related potassium channel; Ca_V_: calcium voltage-gated channel; HCN: hyperpolarization-activated cyclic nucleotide-gated channel; TRPV: transient receptor potential vanilloid;TRPM2 transient receptor potential melastatin 2; CMS: chronic mild stress; aLH: acute learned helplessness; CSDS: chronic social defeat stress; CUMS: chronic unpredictable mild stress; WKY: Wistar Kyoto; cLH: congenitally learned helpless; LPS: lipopolysaccharide; CSI: chronic social Isolation; CRS: chronic restraint stress; CUS: chronic unpredictable stress; SDS: social defeat stress; SNI: spared nerve injury; FC: frontal cortex; PFC: prefrontal cortex; LHb: lateral habenula; LHA:lateral hypothalamic area; NAc: nucleus accumbens; VTA ventral tegmental area; MHb: medial habenula; mPFC: medial prefrontal cortex; DRN: dorsal raphe nucleus

Animal studies reveal that *KCNB1* mRNA, encoding K_V_2.1, is upregulated in the lateral habenula (LHb) of mice exhibiting acute learned helplessness [51]. Chronic mild stress (CMS) induces changes in the expression of K_V_2.1 and K_V_4.2 in the frontal cortex and hippocampus, with fluoxetine treatment reversing only the K_V_2.1 changes [52]. Chronic social defeat stress (CSDS) decreases the expression of K_V_4.2 in the lateral hypothalamic area GABAergic neurons and K_V_7.4 in the ventral tegmental area (VTA) dopaminergic neurons [53, 54], with functional degradation of K_V_4.2 also observed in the nucleus accumbens (NAc) medium spiny neurons of mice subjected to chronic unpredictable mild stress (CUMS) [55]. Kv4.2 knockout (KO) mice show increased immobility during forced swimming, and medial prefrontal cortex (mPFC) layer 5 pyramidal neurons receiving 5-hydroxytryptamine (5-HT) have a reduced increase in spontaneous excitatory postsynaptic currents (sEPSCs) frequency after a single swimming stress compared to wild-type mice [56].

Elevated levels of Kir4.1 protein in astrocytes of the LHb and hippocampus are found in congenitally learned helpless and lipopolysaccharide (LPS) models [57, 58]. Increased expression of *KCNJ9* (Kir3.3) and *KCNJ5* (Kir3.4) is observed in the medial habenula and LHb of Wistar-Kyoto rats [59]. Chronic stress raises Kir6.1 and Kir6.2 expression in the hippocampus [60], but not in the mPFC [61]. Astrocyte conditional knockout (cKO) of Kir4.1 impairs the dynamic balance of extracellular potassium ions and glutamate (Glu), and reduces the amplitude and frequency of sEPSCs in CA1 pyramidal neurons of Kir4.1 cKO mice [62]. Genetic deletion of Kir6.1 increases the frequency of sEPSCs in hippocampal CA3 pyramidal neurons [63].

Chronic stress and LPS enhance TWIK-related potassium channel (TREK) 1 expression in the hippocampus and frontal cortex [52, 64, 65]. TREK1 deficient mice exhibit resistance to depression, enhancing 5-HT neurotransmission efficacy and reducing corticosterone levels under stress [66]. Specific knockdown of TREK1 in mouse hippocampal neurons increases the amplitude of miniature excitatory postsynaptic currents (mEPSCs) in hippocampal CA1 pyramidal neurons and attenuates CUMS-induced reduction in mEPSCs amplitude and depressive-like behavior. Conversely, specific overexpression of TREK1 in hippocampal neurons promotes CUMS-induced decreases in CA1 pyramidal mEPSCs amplitude and exacerbates depressive-like behavior in mice [64].

In rats subjected to chronic restraint stress (CRS), hippocampal pyramidal neurons exhibit elevated expression of the L-type voltage-gated calcium channel subunit alpha-1C (Ca_V_1.2) at both the mRNA and protein levels, accompanied by an enhanced amplitude of L-type calcium currents [67]. Conversely, chronic unpredictable stress (CUS) induces a delayed upregulation of Ca_V_1.2 protein expression specifically within the PFC, a phenomenon not observed in other stress-responsive brain regions such as the hippocampus or amygdala [68]. The *CACNA1C* gene, which encodes Ca_V_1.2, is pivotal in regulating the intracellular second messenger system, thereby influencing synaptic plasticity, gene expression, and neurotransmitter release. Studies have demonstrated that hippocampal synaptic plasticity is compromised in mice with a conditional knockout of *CACNA1C* in the hippocampus [69]. In these Ca_V_1.2 cKO mice, the frequency of spontaneous inhibitory postsynaptic currents (sIPSCs) in lateral amygdala principal neurons increases, while the frequency and amplitude of sEPSCs decrease, indicating a shift in the balance of inhibitory and excitatory activity within the lateral amygdala [70].

The expression of the hyperpolarization-activated cyclic nucleotide-gated channel (HCN) 2 is reduced in the VTA of CMS mice and in the NAc Shell of SDS mice [71, 72]. Altered HCN2 expression has been shown to affect neuronal firing frequency, with HCN2 knockdown leading to a significant increase in GABAergic output from reticular thalamic nucleus neurons to ventrobasal neurons [71, 73]. Additionally, the expression of the transient potential receptor vanilloid (TRPV) 2 is diminished in the hippocampus of CUMS rats [74], while TRPV4 and TRPM2 levels are elevated in the hippocampus of LPS and CUS mice [50, 75]. Notably, TRPM2 KO mice exhibit a significant increase in the amplitude and frequency of mEPSCs in hippocampal dentate gyrus neurons, alongside antidepressant-like behavior [50]. These findings collectively suggest that ion channel alterations significantly impact synaptic homeostasis within neural circuits, positioning them as promising targets for the development of novel antidepressant therapies.

## Modulation of ion channels influences depression-like behaviors

The modulation of ion channels has been demonstrated to significantly influence depression-like behaviors in both clinical populations and animal models. For instance, in vitro and in vivo studies have shown that KCNQ-type K + channel openers, when applied to the VTA of mice subjected to social frustration stress, can reduce depressive-like behaviors and mitigate the overactivation of VTA dopamine (DA) neurons [79, 80]. Moreover, the conditional knockout of the multifunctional protein p11 (also known as S100A10) in parvalbumin neurons results in decreased hippocampal K_V_3.1 expression, impairing the high-frequency firing capacity of these neurons and increasing susceptibility to depression. This decrease in presynaptic K_V_3.1 expression leads to enhanced GABAergic synaptic responses, uncontrolled synaptic vesicle release, and disruption of short-term synaptic plasticity in the parvalbumin-granule cell synapses of the dentate gyrus [81]. Clinical research has indicated that Ezogabine, a KCNQ2/3 channel opener, is effective in treating depressive disorders [82]. Additionally, Lys05, a Kir4.1 inhibitor, has shown rapid antidepressant effects in Kir4.1-driven depressive-like phenotypes and various animal models of depression, highlighting Kir4.1 as a potential target for rapid-acting antidepressants [83]. The peptide spadin, which blocks TREK1 channels, has been found to exert antidepressant effects within a short timeframe [84], and N-[4]-N-(2-(3,4-dihydroisoquinolin-2(1H)-yl)−2-oxoethyl)methanesulfonamide (TKDC), an inhibitor of TREK1 channels, exhibits antidepressant-like actions following both acute and chronic treatment [85]. Furthermore, intermediate states of the TREK1 channel are being considered as potential targets for antidepressant therapy [86]. Escitalopram has been reported to inhibit the current of Nav1.2 and alter its activation and inactivation states [87]. In rats, chronic stress reduces hippocampal TRPV2 expression, and the TRPV2 agonist probenecid can alleviate depressive-like behaviors and increase hippocampal levels of 5-HT, norepinephrine (NE), and DA [74]. The deletion of transient receptor potential channel 5 (Trpc5) in oxytocin neurons of the hypothalamic paraventricular nucleus leads to obesity and postnatal depressive behaviors in female mice, whereas overexpression of Trpc5 reverses these phenotypes [88]. Chronic social isolation has been found to upregulate the small-conductance Ca^2+^ activated K + channel 3 (SK3) in the dorsal raphe nucleus, resulting in reduced 5-HT neuronal activity. Inhibitors of SK channels can ameliorate the behavioral deficits caused by chronic social isolation [76]. In CUS-exposed rats, there is an increase in the protein expression of HCN1 and perisomatic *Ih* currents in neurons of the dorsal CA1 region. Administration of shRNA-HCN1 to reduce *Ih* in dorsal CA1 neurons has been shown to mitigate the depressive-like behavioral deficits induced by CUS [77].

## Presynaptic neurotransmitter release in the context of depression

Presynaptic homeostatic plasticity plays a critical role in counterbalancing impaired postsynaptic neurotransmitter receptor function by rapidly and precisely modulating neurotransmitter release [89]. Neurotransmitters, essential endogenous signaling molecules, facilitate communication within the central and peripheral nervous systems [90]. The soluble N-ethylmaleimide-sensitive factor attachment protein receptor (SNARE) complex is central to the presynaptic release of neurotransmitters. This complex is formed through the interaction of synaptic vesicle fusion proteins and synaptic membrane fusion proteins, mediating the fusion of synaptic vesicles with the presynaptic membrane and the subsequent release of neurotransmitters into the synaptic cleft [91–93].

Stress and depression have been shown to influence the expression of the SNARE complex in the brain. Elevated levels of SNARE complex proteins have been observed in the frontal cortex of individuals with suicidal schizophrenia and depression [94]. Additionally, acute foot shock stress in rats has been found to cause the accumulation of presynaptic SNARE complexes in the prefrontal/frontal cortex [95]. Studies on antidepressants have demonstrated that prolonged treatment can reduce depolarization-induced Glu release from hippocampal synaptic terminals, alter protein–protein interactions, modulate the assembly of presynaptic SNARE complexes, and decrease synaptic vesicle fusion and the number of complexes in the presynaptic membrane [96, 97]. For instance, fluoxetine has been shown to impair SNARE complex function, thereby decreasing the release of both Glu and GABA [98].

Depression and stress disrupt the function of both excitatory glutamatergic and inhibitory GABAergic circuits in the brain, compromising the efficiency and integrity of neural networks involved in emotion and cognition [99]. There is increasing evidence that chronic stress-induced anxiety and depression are associated with an imbalance in excitation/inhibition within the PFC [100, 101]. Emerging research indicates that synaptic transmission of both excitatory and inhibitory presynaptic neurotransmitters is altered during depressive states (see Table 2), particularly in terms of the probability of presynaptic neurotransmitter release and changes in synaptic vesicle volume and quantity. For example, CSDS significantly increases the paired-pulse ratio (PPR) of excitatory postsynaptic currents in basolateral amygdala (BLA) synapses projecting to the mPFC and the ventral hippocampus, indicating reduced Glu release probability [102]. In a learned helplessness model, the frequency of mEPSCs was decreased, and the PPR was elevated in the ventrolateral periaqueductal gray region of the midbrain in rats [103]. Chronic restraint stress in mice reduced the frequency of miniature inhibitory postsynaptic currents (mIPSCs) in layers II/III of the anterior cingulate cortex (ACC) and increased the PPRs of field excitatory postsynaptic potentials and evoked excitatory postsynaptic currents [104]. Conversely, chronic stress decreased the frequency of mIPSCs in parvocellular neurons of the hypothalamic paraventricular nucleus without affecting the PPR, suggesting a reduction in the number of presynaptic GABAergic synapses [105]. Acute stress enhances both the readily releasable pool of vesicles and depolarization-evoked Glu release [106], while chronic stress alters the distribution pattern of synaptic vesicles and increases vesicle density in the CA3 region of the hippocampus [107]. Furthermore, chronic stress has been proposed to reduce the number of synaptic vesicles in the inner molecular layer of the hippocampal dentate gyrus [108]. In summary, presynaptic neurotransmitter release in neural circuits is significantly altered during stress and depression, primarily through changes in the probability of neurotransmitter release and modifications to the synaptic vesicle pool.

**Table 2:** Alterations in presynaptic excitatory and inhibitory neurotransmission in depression

Synaptic transmission	Animal Model	Brain region	Frequency change	References
sEPSC	RS rats	DRN	Increase	[109]
	HFD + CSDS mice	mPFC	Decrease	[110]
	CVS mice	PVN	Increase	[111]
	CIS mice	mPFC	Decrease	[112]
mEPSC	CUS mice	mPFC PrL D1-PYR/ D2-PYR	Increase/ Decrease	[113]
	ELS mice	mPFC IL	Decrease	[114]
	LH rats	vlPAG	Decrease	[103]
	CUS rats	mPFC	Decrease	[115]
	LANs rats	vlPAG	Decrease	[116]
	ELS mice	mPFC IL	Decrease	[117]
sIPSC	HFD + CSDS mice	mPFC	Decrease	[110]
	CSDS mice	mPFC-LHb neurons	Decrease	[118]
	CUMS mice	NAC	Decrease	[119]
	CUMS mice	Prelimbic cortical	Decrease	[120]
	SDPS rats	Hippocampus CA1	Decrease	[121]
mIPSC	CSDS mice	NAC	Decrease	[122]
	long-term isolation mice	Intracentral amygdala	Decrease	[123]
	CRS mice	Hippocampus	Decrease	[124]
	CVS mice	mPFC	Increase	[125]
	CRS mice	ACC	Decrease	[104]
	CUS mice	mPFC PrL D1-PYR	Increase	[113]
	MS rats	Hippocampus CA1 pyramidal cells	Increase	[126]
	ELA mice	NAc medium spiny neurons	Decrease	[127]
	RMS mice	Hippocampus CA1	Decrease	[128]

sEPSC: spontaneous excitatory postsynaptic currents; mEPSC: miniature excitatory postsynaptic currents; sIPSC: spontaneous inhibitory postsynaptic currents; mIPSC: miniature inhibitory postsynaptic currents; RS: restraint stress; HFD + CSDS: high-fat diet + chronic social defeat stress; CVS: chronic variable stress; CIS: chronic immobilization stress; CUS:chronic unpredictable stress; ELS: early life stress; LH: learned helplessness; LANs: lights at night; CSDS: chronic social defeat stress; CUMS: chronic unpredictable mild stress; SDPS: social defeat-induced persistent stress; CRS: chronic restraint stress; MS: maternal separation stress; ELA: early life adversity; RMS: repeated maternal separation; DRN: dorsal raphe nucleus; PVN: paraventricular nucleus; mPFC: medial prefrontal cortex; PrL: prelimbic cortex; IL: infralimbic cortex;D1-PYR: dopamine D1-expressing pyramidal neurons; D2-PYR: dopamine D2-expressing pyramidal neurons; vlPAG: ventrolateral periaqueductal gray; NAc: nucleus accumbens; ACC: anterior cingulate cortex

Neurotransmitter release involves several pivotal processes: (1) metabolism by enzymes, (2) reuptake by the presynaptic neuron, and (3) binding to receptors on postsynaptic neurons or target cells, thereby triggering a physiological response in the postsynaptic or adjacent cell. Disruptions in neurotransmitter release can alter local neurotransmitter concentrations, leading to impairments in brain function that contribute to a range of physical, psychiatric, and neurodegenerative disorders [129]. Extensive research has established a strong correlation between neurotransmitter levels and the incidence of depression [130, 131]. Studies have demonstrated that specific neurotransmitters—such as 5-HT, Glu, GABA, NE, and DA—are essential for maintaining normal mood and are implicated in the pathogenesis of depression [132–135]. Notably, patients with MDD exhibit significantly elevated serum glutamate levels compared to controls, suggesting a potential link between Glu alterations and MDD [136]. Cerebrospinal fluid analyses in depressed individuals have revealed that 5-HT levels are associated with the onset of depressive symptoms [137], and pre-treatment cerebrospinal fluid Glu levels in depressed patients have been found to correlate positively with suicidal ideation [138]. Stress has been shown to influence DA levels in the PFC and NAc [139, 140]. Moreover, various antidepressant treatments achieve their therapeutic effects by modulating neurotransmitter levels [141, 142].

## Synaptic scaling of the postsynaptic density in the context of depression

The postsynaptic density (PSD) is a specialized region of the cytoskeleton at the synaptic junction, serving as the structural foundation for postsynaptic signaling and integration [143–145]. The outer surface of the PSD is rich in neurotransmitter receptors involved in homeostatic synaptic scaling, as well as trans-synaptic adhesion molecules embedded in the plasma membrane [146]. Autopsy studies have revealed significantly decreased expression of the PSD marker protein PSD-95 in the PFC of individuals with depression [147]. Animal studies have shown that six weeks of exposure to CUMS results in reduced levels of brain-derived neurotrophic factor (BDNF), synaptophysin, and PSD-95, along with ultrastructural changes such as decreased synaptic number density, surface density, and PSD thickness in lateral amygdala neurons [148].

**Fig. 2:**
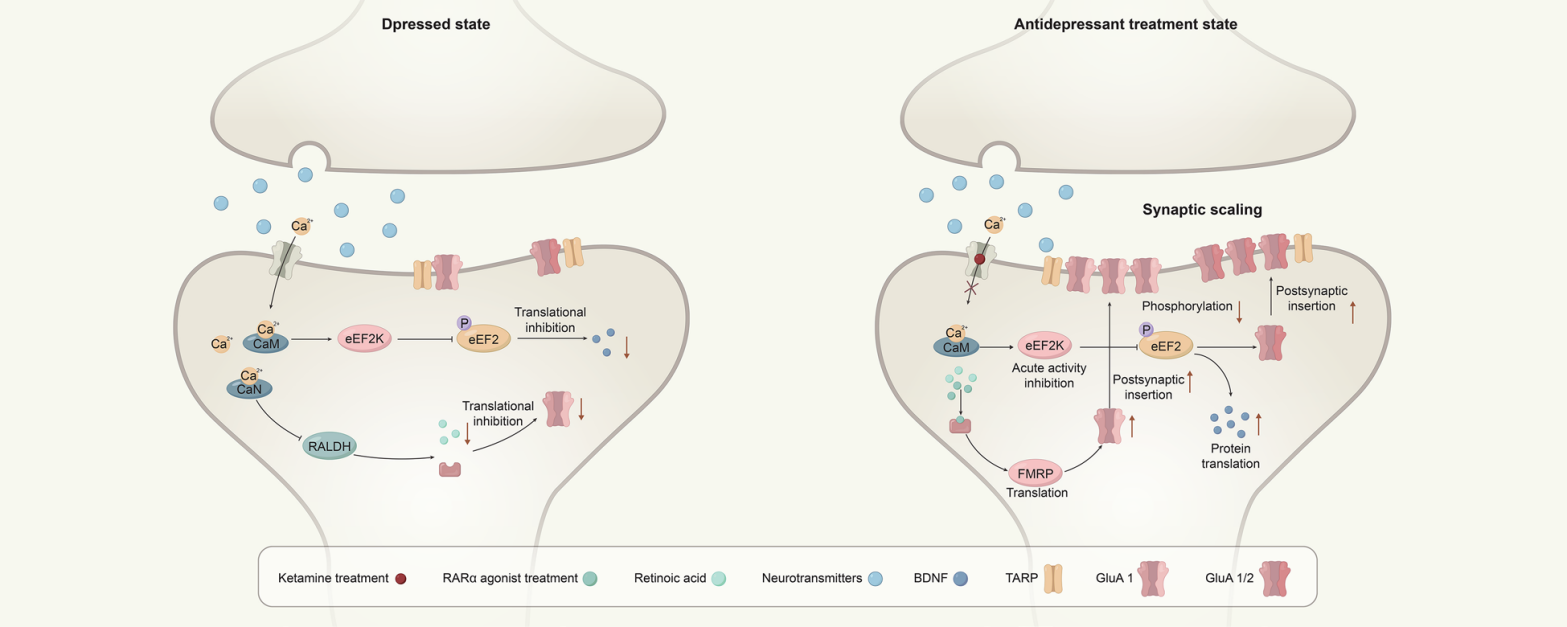
Ketamine and RARα receptor agonists can regulate synaptic scaling through different pathways and exert antidepressant effects. A simplified overview of the antidepressant effects of ketamine and RARα receptor agonists through the regulation of synaptic scaling. Ketamine enhances the recruitment of postsynaptic GluA1/2, facilitating synaptic scaling and driving rapid antidepressant effects. Similarly, RARα receptor agonists directly activate retinoic acid receptors, which also leads to an increased recruitment of postsynaptic GluA1, thereby promoting synaptic scaling. This mechanism produces antidepressant effects comparable to those of ketamine. *eEF2K* elongation factor 2 kinase, *eEF2* elongation factor 2, *FMRP* fragile X mental retardation protein, *CaM* calmodulin, *CaN* calcineurin, *RALDH* retinal dehydrogenase, *BDNF* brain-derived neurotrophic factor, *GluA1/2* AMPA receptor subunits 1/2

Homeostatic regulation of postsynaptic neurotransmitter receptors, termed synaptic scaling, involves the bi-directional regulation of the amplitude of mEPSCs to counterbalance chronic alterations in neuronal activity [149, 150]. An increase in firing rate is met with a proportional decrease in excitatory synaptic strength, while a decrease in firing rate leads to a proportional increase in synaptic strength [151]. These changes in synaptic strength are mediated by adjusting the quantity of α-amino-3-hydroxy-5-methyl-4-isoxazole propionic acid receptors (AMPARs) in the postsynaptic membrane [152]. Synaptic scaling continuously modulates the number of receptors in the postsynaptic membrane through mechanisms involving receptor trafficking, expression of scaffolding proteins, gene transcription, and neural activity modulation [149, 153].Both retinoic acid receptor α (RARα) agonists, such as AM580, and ketamine modulate synaptic scaling by affecting the translation of AMPAR proteins in the hippocampus, thereby exerting antidepressant effects through distinct pathways [154, 155] (Fig. 2). The eukaryotic elongation factor 2 (eEF2) signaling pathway is a key regulator of protein synthesis, synaptic plasticity, learning, and memory [156]. Ketamine induces rapid antidepressant-like effects by inhibiting spontaneous N-methyl-D-aspartate receptor (NMDAR)-mediated mEPSCs, leading to acute inhibition of eEF2 kinase activity and a subsequent rapid increase in BDNF translation. Ketamine’s synaptic scaling is associated with upregulation of the AMPAR subunits GluA1 and GluA2 [157]. The RARα agonist AM580 can elicit a ketamine-like rapid antidepressant response through RARα activation, with its synaptic scaling achieved by the insertion of AMPARs containing the GluA1 subunit [158, 159].

## Related signaling molecules in the regulation of synaptic homeostasis and their relevance to depression

In the field of depression research, the importance of various signaling molecules in maintaining synaptic homeostasis has gained significant attention. This section highlights the roles of three key molecules: a neurotrophic factor that enhances neuronal communication, a cytokine released by glial cells with profound neuronal effects, and a small molecule derived from Vitamin A metabolism.

### Brain-derived neurotrophic factor (BDNF)

BDNF is a vital neurotrophic factor essential for the development and regulation of synaptic plasticity [160]. Upon release, BDNF binds to its high-affinity receptor, tropomyosin receptor kinase B (TrkB), initiating a series of synaptic modulation events. BDNF plays a central role in synaptic homeostasis by influencing neurotransmitter release from presynaptic terminals. For instance, a study using viral-mediated gene targeting in the CA3-CA1 hippocampal circuit demonstrated that the absence of presynaptic TrkB reduced the probability of neurotransmitter release, underscoring the critical role of presynaptic TrkB receptors in BDNF-mediated presynaptic function [161]. Additionally, BDNF modulates the density of postsynaptic receptors. Research on the antimanic effects of lithium revealed that chronic lithium treatment decreased the surface expression of the GluA1 subunit in hippocampal neurons, significantly reducing AMPAR-mediated mEPSCs. This effect was mediated by BDNF and TrkB, highlighting their importance in postsynaptic homeostatic plasticity [162]. The link between BDNF and depression is well-documented. Duman and Monteggia proposed the neurotrophic hypothesis of depression, which posits that stress and depression lead to a deficiency in neurotrophic factors, contributing to cellular atrophy and loss in key brain regions of individuals with MDD [163]. Studies have consistently shown that MDD patients exhibit lower levels of central and peripheral BDNF compared to non-depressed individuals, with peripheral BDNF levels inversely correlated with symptom severity and directly associated with symptom improvement [164, 165]. Innovative approaches to BDNF delivery have shown promise. For example, a study utilizing quercetin nanogels to deliver BDNF demonstrated that BDNF-quercetin alginate nanogels could effectively cross the blood–brain barrier via the nasal-brain route, providing sustained and controlled BDNF release in the brain. Treatment with these nanogels significantly increased plasma and hippocampal BDNF levels in rats subjected to CUMS, potentially exerting antidepressant effects through the regulation of the glutamatergic system, the phosphoinositide 3-kinase-Protein kinase B (PI3K-Akt) pathway, and the BDNF-TrkB signaling pathway [166].Furthermore, ketamine has been shown to rapidly induce BDNF protein synthesis by inhibiting eukaryotic elongation factor 2 (eEF2) kinase, leading to increased surface expression of the GluA1 and GluA2 subunits. This regulation of synaptic homeostasis contributes to ketamine's rapid antidepressant-like effects [155, 157]. These findings collectively suggest that the rapid upregulation of proteins such as BDNF can modulate synaptic homeostasis to mediate antidepressant responses.

### Tumor necrosis factor-alpha (TNF-α)

TNF-α, a key cytokine in the CNS, plays a dual role in maintaining synaptic homeostasis and contributing to the pathogenesis of depression [167]. Glia-derived TNF-α is crucial for the synaptic scaling of both excitatory and inhibitory synapses. Research has demonstrated that prolonged neuronal inactivity triggers the release of soluble TNF-α from glial cells. This accumulation of TNF-α enhances AMPA receptor levels at excitatory synapses while downregulating GABA receptor levels at inhibitory synapses in a homeostatic manner that depends on TNF-α receptors. Notably, Hebbian forms of synaptic plasticity do not require TNF-α [168]. However, some studies suggest that glia-derived TNF-α may not directly drive synaptic scaling but is essential for maintaining synaptic plasticity in a stable state [169].

Clinical evidence supports the involvement of TNF-α in depression. A meta-analysis of 24 studies revealed that plasma TNF-α levels are significantly elevated in individuals with depression compared to healthy controls [170]. Additionally, the protein and mRNA levels of TNF-α in the PFC of depressed individuals who died by suicide were markedly higher than in control groups [171]. Clinical studies have also shown that reductions in peripheral TNF-α levels correlate with improvements in depressive symptoms, and effective treatment for MDD normalizes TNF-α levels [172, 173]. The surge in cytokine release (including TNF-α, interleukin-1 beta and interleukin-6) during early life inflammation leads to dysregulation of microglial phagocytic capacity. During adolescence, unpredictable stressors exacerbate microglial phagocytosis around the spines of glutamatergic neurons in the ACC, promoting depressive-like behaviors [174]. In animal models, wild-type mice treated with antidepressants fluoxetine and desipramine showed reduced immobility in the forced swim test and tail suspension test. In contrast, TNF-α KO mice exhibited a diminished response to these medications, requiring higher doses to achieve an antidepressant effect. Furthermore, selective ablation of TNF-α in astrocytes confirmed that astrocytic TNF-α is essential for the antidepressant effects of chronic fluoxetine treatment [175].

### Retinoic acid (RA)

RA, a metabolite of vitamin A, is well-known for its role in embryonic development but also significantly influences synaptic homeostasis and plasticity in the adult brain [176]. In hippocampal cultures, suppression of neuronal activity with tetrodotoxin (TTX) and APV induced synaptic scaling, which was associated with increased RA synthesis. Inhibition of RA synthesis abolished this synaptic scaling. This process is mediated by RARα signaling, which promotes local synthesis of the GluA1 subunit. Knockdown of RARα blocks RA-induced synaptic scaling, while activation of RARα receptors replicates the effects of RA on synaptic scaling [158].RA has been implicated in depressive disorders [177]. Preclinical and epidemiological studies indicate that serum retinol levels are significantly elevated in MDD patients compared to healthy individuals, and the synthesis of the vitamin A metabolite all-trans RA is enhanced, suggesting a critical role for the RA system in depression [178]. Chronic RA treatment can induce depressive- and anxiety-like behaviors by stimulating GABA synthesis, increasing GABA receptor expression, and downregulating glutamate receptor expression. These changes reduce hippocampal neuronal excitability and may impair hippocampal homeostatic synaptic plasticity by lowering GluA1 mRNA levels [179]. Conversely, acute activation of the RA signaling pathway has been shown to induce synaptic scaling in hippocampal neurons and mediate antidepressant-like effects in mice [155]. Thus, RA is a key regulator of synaptic homeostasis and plays a significant role in depression.

The network of signaling molecules involved in synaptic homeostasis is extensive and not yet fully understood. For example, molecules such as Endostatin, Presenilin 1, and Arc/Arg3.1 have been reported to regulate synaptic homeostasis [180–182], but their precise links to synaptic homeostasis in depression remain to be fully elucidated.

## Conclusion

The array of mechanisms involved in synaptic homeostasis is extensive, including ion channels, presynaptic neurotransmitter release, postsynaptic receptors, and related signaling molecules. However, their precise connections to synaptic homeostasis in the context of depression remain to be fully understood. Therefore, a deeper understanding of the molecular mechanisms governing synaptic homeostasis is essential for clarifying the links between synaptic homeostasis and depression. Moreover, the etiology and therapeutic response in depression are intricately linked to disruptions in synaptic homeostasis within mood-regulating brain regions and circuits. Current investigations into the impact of stress and depression, as well as the synaptic effects of antidepressant therapies, have primarily focused on the PFC and hippocampus. However, emerging evidence indicates that other brain regions and circuits, such as those involved in reward and anti-reward processing (e.g., VTA, NAc, and LHb), also play significant roles in the pathophysiology of depression. Future research is essential to elucidate the effects of antidepressant treatments on synaptic homeostasis across additional circuits and neuronal populations. Together, this review contributes to understanding the implications of altered synaptic homeostasis on depression, which paves the way for the development of more accessible, safer, and efficacious antidepressant interventions.

## Data Availability

Not applicable.

## Abbreviations

CNS: Central nervous system
SNARE: Soluble N-ethylmaleimide-sensitive factor attachment protein receptor
PFC: Prefrontal cortex
LHb: Lateral habenula
VTA: Ventral tegmental area
NAc: Nucleus accumbens
mPFC: Medial prefrontal cortex
BLA: Basolateral amygdala
ACC: Anterior cingulate cortex
Glu: Glutamate
GABA: γ-Aminobutyric acid
NE: Norepinephrine
5-HT: 5-Hydroxytryptamine
DA: Dopamine
AMPARs: α-Amino-3-hydroxy-5-methyl-4-isoxazole propionic acid receptors
GluA1/2: AMPA receptor subunits 1/2
RA: Retinoic acid
RARα: Retinoic acid receptor α
NMDAR: N-methyl-d-aspartate receptor
eEF2: Eukaryotic elongation factor 2
TrkB: Tropomyosin receptor kinase B
PI3K: Phosphoinositide 3-kinase
Akt: Protein kinase B
TNF-α: Tumor necrosis factor-alpha
MDD: Major depressive disorder
K_V_: Voltage-gated potassium channel
Kir: Inwardly rectifying potassium channel
TRPM2: Transient receptor potential melastatin 2
TREK: TWIK-related potassium channel
SK3: Small-conductance Ca^2^^+^ activated K^+^ channel 3
Ca_V_1.2: L-type voltage-gated calcium channel subunit alpha-1C
HCN: Hyperpolarization-activated cyclic nucleotide-gated channel
TRPV: Transient potential receptor vanilloid
Trpc5: Transient receptor potential channel 5
Na_V_: Voltage-gated sodium channel
PSD: Postsynaptic density
BDNF: Brain-derived neurotrophic factor
CMS: Chronic mild stress
CSDS: Chronic social defeat stress
CUMS: Chronic unpredictable mild stress
LPS: Lipopolysaccharide
CRS: Chronic restraint stress
CUS: Chronic unpredictable stress
PPR: Paired-pulse ratio
mEPSCs: Miniature excitatory postsynaptic currents
mIPSCs: Miniature inhibitory postsynaptic currents
sEPSCs: Spontaneous excitatory postsynaptic currents
sIPSCs: Spontaneous inhibitory postsynaptic currents
cKO: Conditional knockout
KO: Knockout
